# Metabolic responses of thermophilic endospores to sudden heat-induced perturbation in marine sediment samples

**DOI:** 10.3389/fmicb.2022.958417

**Published:** 2022-08-12

**Authors:** Anirban Chakraborty, Jayne E. Rattray, Sienna S. Drake, Stuart Matthews, Carmen Li, Bo Barker Jørgensen, Casey R. J. Hubert

**Affiliations:** ^1^Department of Biological Sciences, Idaho State University, Pocatello, ID, United States; ^2^Department of Biological Sciences, University of Calgary, Calgary, AB, Canada; ^3^Section for Microbiology, Department of Biology, Aarhus University, Aarhus, Denmark

**Keywords:** thermophiles, endospores, dormancy, metabolomics, sediment microbiome

## Abstract

Microbially mediated processes in a given habitat tend to be catalyzed by abundant populations that are ecologically adapted to exploit specific environmental characteristics. Typically, metabolic activities of rare populations are limited but may be stimulated in response to acute environmental stressors. Community responses to sudden changes in temperature and pressure can include suppression and activation of different populations, but these dynamics remain poorly understood. The permanently cold ocean floor hosts countless low-abundance microbes including endospores of thermophilic bacteria. Incubating sediments at high temperature resuscitates viable spores, causing the proliferation of bacterial populations. This presents a tractable system for investigating changes in a microbiome's community structure in response to dramatic environmental perturbations. Incubating permanently cold Arctic fjord sediments at 50°C for 216 h with and without volatile fatty acid amendment provoked major changes in community structure. Germination of thermophilic spores from the sediment rare biosphere was tracked using mass spectrometry-based metabolomics, radiotracer-based sulfate reduction rate measurements, and high-throughput 16S rRNA gene sequencing. Comparing community similarity at different intervals of the incubations showed distinct temporal shifts in microbial populations, depending on organic substrate amendment. Metabolite patterns indicated that amino acids and other sediment-derived organics were decomposed by fermentative *Clostridia* within the first 12–48 h. This fueled early and late phases of exponential increases in sulfate reduction, highlighting the cross-feeding of volatile fatty acids as electron donors for different sulfate-reducing *Desulfotomaculia* populations. The succession of germinated endospores triggered by sudden exposure to high temperature and controlled by nutrient availability offers a model for understanding the ecological response of dormant microbial communities following major environmental perturbations.

## Introduction

The microbiome of any habitat is comprised of active and inactive organisms, with the abundance of inactive populations often rivaling or exceeding the active members (Lennon and Jones, 2011). Microbially mediated processes within an ecosystem are driven by active populations physiologically equipped and ecologically adapted to exploit prevailing environmental conditions. Acute disturbances, including flooding (Francioli et al., 2021), toxic chemical spills (Joye et al., 2014), or sudden increases in temperature due to, for example, forest fires (Lee et al., 2017) or hot hydrothermal vent fluids mixing in cold seawater (Dick, 2019), can be major drivers of ecological dynamics by introducing unpredictable spatiotemporal changes in ecosystem composition and functioning. While the effects of such dramatic events are often deleterious to active abundant populations, they can offer a selective advantage to rare inactive or dormant organisms, allowing new populations to gain a foothold in the community (Sorensen and Shade, 2020). Investigating microbiome community succession triggered by major perturbations can therefore deliver a better understanding of microbiome responses to ecological disturbance.

Marine sediments host a vast and diverse microbiome (Gibbons et al., 2013; Hoehler and Jørgensen, 2013; Orsi, 2018) including inactive bacterial endospores that are widespread throughout the oceans (Müller et al., 2014). The marine subsurface has recently been reported to host a surprisingly high abundance of dormant endospores, with estimates suggesting there are up to >10^29^ endospores within the uppermost kilometer of marine sediment globally. This supports the hypothesis that the biomass contributed by the endospores in the subsurface may even surpass that of vegetative cells in this vast environment (Lomstein et al., 2012; Wörmer et al., 2019; Heuer et al., 2020). The unique ability of spore-forming bacteria to persist in a reversible state of metabolic inactivity coupled with the exceptionally resistant structure of endospores allows preservation of viability under extreme environmental stress (Reineke et al., 2013; O'Sullivan et al., 2015). Endospores are not affected by natural selection in the way that active, vegetative populations within the marine microbiome are (de Rezende et al., 2013) thus making spores good candidates for passive spreading from one place to another aided by environmental vectors such as ocean currents (Müller et al., 2014) or geofluids (Chakraborty et al., 2018; Gittins et al., In Press).

Discoveries of viable endospores of anaerobic thermophilic bacteria in permanently cold seabed sediments in the Arctic and elsewhere underscore the prevalence of “misplaced microbes” in the sedimentary biosphere (Bartholomew and Paik, 1966; Isaksen et al., 1994; Hubert et al., 2009). DNA-based biodiversity surveys generally do not include the portion of the microbiome that exists as endospores, most likely due to endospore resistance to physical or chemical lysis steps employed during community DNA extraction from environmental samples (Bueche et al., 2013; Wunderlin et al., 2014). Laboratory incubations have been successfully implemented for investigating the physiology, diversity, abundance, and distribution of endospore-forming bacteria in seawater and marine sediments (Hubert et al., 2010; de Rezende et al., 2013, 2017; Nielsen et al., 2017; Volpi et al., 2017; Bell et al., 2018; Chakraborty et al., 2018; Cramm et al., 2019). Some of these studies have showcased the metabolic versatility displayed by these thermophilic populations, including organic matter mineralization by obligately fermentative bacteria (Hubert et al., 2010; Volpi et al., 2017) and sulfate reduction by members of the *Desulfotomaculia* (Hubert et al., 2010; Cramm et al., 2019; Bell et al., 2020).

Incubation-based spore investigations depend on a multitude of factors such as the temperature, pressure and nutrients, spore abundance in the sample inoculum, sample storage conditions, and sediment heterogeneities (Hubert et al., 2010; Cramm et al., 2019; Bell et al., 2020). Furthermore, sporulation and germination are complex stepwise processes that can be temporally variable between diverse spore-forming taxa (Setlow, 2003; Paredes-Sabja et al., 2011; Tocheva et al., 2016). The length of incubation and sub-sampling frequency can determine the diversity of germinating taxa. Metabolite profiles characterizing germinating spore populations have focused on the targeted analysis of compounds of interest; fluorescently labeled polysaccharides have been used to assess complex macromolecular substrate degradation (Hubert et al., 2010), while HPLC methods have focused on small organic acids (Hanson et al., 2019). In this study, high-temperature incubations of cold marine sediments featuring high-frequency subsampling and 16S rRNA gene amplicon sequencing were used to investigate heat-induced community succession and metabolism by germinating endospore populations. Cross-feeding between disparate metabolic groups was investigated in detail by combining untargeted Orbitrap mass-spectrometry analyses with radiotracer measurements of sulfate reduction rates (SRRs) for the first time. The observations offer a useful model for a microbiome response to ecosystem disturbance and highlight intricacies of microbial succession and associated metabolite turnover in response to sudden and dramatic environmental change.

## Materials and methods

### Sampling of marine sediments

Marine surface sediments (3–9 cm below sea floor) were collected from Smeerenburgfjorden (Station J; 79°56′N, 11°05′E), situated on the north-west coast of the Svalbard archipelago during multiple research expeditions conducted between 2003 and 2007 (Hubert et al., 2010). A Haps corer (Kanneworff and Nicolaisen, 1983) fitted with a coring tube was used to collect sediments from the middle of the fjord where the water depth is 210 m and sediment temperature at the time of collection was −2 to +2°C (measured using a digital probe). Sediment samples for microbiological analyses were sealed in foil or plastic bags with minimum air exposure and stored at 4°C.

### Incubation of pasteurized sediment slurries at 50°C

Non-homogenized cold sediments (ca. 10 g) were directly added to sterile serum bottles inside a walk-in cold room (4°C). Serum bottles were immediately sealed with sterile rubber stoppers and the headspace was exchanged with N_2_:CO_2_ (90:10%). Sediment aliquots were subsequently diluted in a 1:2 (w/w) ratio with an anoxic, artificial seawater medium (Isaksen et al., 1994) amended with sulfate under a constant flow of N_2_:CO_2_. To minimize competition for limited substrates between different microbial groups (e.g., sulfate-reducing and fermentative bacteria), subsets of slurries were amended with a combination of six low-molecular-weight volatile fatty acids (VFAs), namely acetate, butyrate, formate, lactate, propionate, and succinate. Concentrations of each of the added VFAs were between 3.88 and 4.45 mM. Parallel sets of slurries received no VFA amendment. All slurries were pasteurized at 80°C for 1 h to eliminate viable vegetative cells and then transferred to 50°C to promote germination and growth of thermophilic endospores. Slurries were incubated for 216 h and were subsampled beginning at time-zero (after pasteurization) and continuing every 3 or 6 or 12 h up to 96 h. After this subsampling occurred every 12–48 h up to 216 h. Slurry subsamples were removed using N_2_:CO_2_-flushed sterile syringes and immediately stored at −20°C until further analysis.

### Analysis of sulfate and volatile fatty acids

Subsamples from all slurries were analyzed for sulfate and VFA concentrations. Slurry subsamples (0.5 ml) were centrifuged at 21,000 × g for 10 min and supernatants were subsequently filtered through syringe filters (0.22 μm pore size) to remove any remaining suspended particles. Sulfate concentrations were measured using a Dionex ICS-5000 reagent-free ion chromatography system (Thermo Scientific, CA, USA) equipped with an anion-exchange column (Dionex IonPac AS22; 4 × 250 mm; Thermo Scientific, CA, USA), an EGC-500 K_2_CO_3_ eluent generator cartridge, and a conductivity detector. An isocratic separation method was used with a constant flow rate of 1.3 ml min^−1^ while maintaining column temperature at 30°C. VFA concentrations were determined using ion-moderated partition chromatography followed by UV detection (210 nm) on a Dionex UltiMate-3000 ultra high-performance chromatography (UHPLC) system (Thermo Scientific, CA, USA) equipped with an Aminex HPX-87H column (9 μm; 7.8 × 300 mm; Bio-Rad, CA, USA). Optimum separation was achieved using 5 mM H_2_SO_4_ as the eluent at a constant flow rate of 0.6 ml min^−1^ and 60°C column temperature, respectively.

### Amplicon sequencing from sediment slurries

Genomic DNA was extracted from subsamples of triplicate sediment slurries representing all time intervals, and from triplicate aliquots of unheated sediments (0d). Equal volumes of slurry from each replicate subsample were pooled and the mixed slurry was used for DNA extraction using the DNeasy PowerLyzer PowerSoil kit (MO BIO Laboratories, a Qiagen Company, Carlsbad, CA, USA). The v3-4 region of the bacterial 16S rRNA gene was amplified using the primer pair SD-Bact-341-bS17/SD-Bact-785-aA21 (Klindworth et al., 2013) modified with Illumina MiSeq overhang adapters. Each PCR reaction consisted of 1 to 2 μl (~20 ng) genomic DNA template, 2.5 μl of each of the primers (final concentration 1 μM), 12.5 μl 2X Kapa HiFi HotStart ReadyMix (Kapa Biosystems, Wilmington, MA, USA), and sterile nuclease-free water to make a final volume of 25 μl. To achieve optimal annealing, a touchdown PCR program was designed, as follows: initial denaturation at 95°C for 5 min, 10 cycles of 95°C for 30 s, 60°C (−1°C/cycle) for 45 s, 72°C for 1 min, followed by 20 cycles of 95°C for 30 s, 55°C for 45 s, 72°C for 1 min, and final extension at 72°C for 5 min. All PCR reactions were performed in triplicate, pooled, and purified using the NucleoMag NGS Clean-up and Size Select kit (Macherey-Nagel Inc., Bethlehem, PA, USA). The purified PCR products were indexed following the instructions on Illumina's 16S amplicon library preparation guide. The concentration of dsDNA and the size of the indexed amplicons were verified using the Qubit dsDNA High Sensitivity assay kit on a Qubit 2.0 fluorometer (Thermo Fisher Scientific, Canada) and the High Sensitivity DNA kit on an Agilent 2100 Bioanalyzer system (Agilent Technologies, Mississauga, ON, Canada), respectively. Indexed amplicons were then pooled in equimolar amounts and sequenced using Illumina's v3 600-cycle (paired-end) reagent kit on a MiSeq benchtop sequencer (Illumina Inc., San Diego, CA, USA).

### Sequence processing and diversity analyses

A total of 1,565,032 demultiplexed paired-end raw reads were processed using the open-source R package DADA2 version 1.10 (Callahan et al., 2016). First, all technical sequences were removed from the raw reads using the Cutadapt tool (Martin, 2011). Based on the quality profile of the raw reads, the forward and the reverse reads were truncated to 250 and 220 nucleotides, respectively. Reads were eliminated if the maximum expected error exceeded 2; reads were also truncated at the first instance in the sequence where the quality score was <2. The quality-controlled forward and reverse reads were further subjected to an Illumina-platform-specific amplicon denoising algorithm in the DADA2 workflow to parse out sequence-specific errors. After filtering out erroneous and singleton sequences, 1,289,685 forward and reverse reads (on average 47,766 reads per library) were subsequently de-replicated and merged followed by the removal of chimeric sequences to generate 3,044 unique ASVs. Taxonomy was assigned to ASVs using the SILVA database version 138 (Quast et al., 2013).

An ASV was considered present in an amplicon library if its relative sequence abundance was at least 0.1%, that is, if at least 24 reads belonged to that ASV within a library containing 24,000 reads. This criterion for defining ASV occurrence was chosen to minimize any influence of platform-specific carry-over contamination during sequencing on the MiSeq platform (Nelson et al., 2014; Dong et al., 2017). If an ASV was absent (<0.1% relative sequence abundance) in the sediment before incubation and was present in >1% relative sequence abundance in at least one amplicon library from all incubated slurries, then that ASV was considered to represent a bacterial population that was enriched during slurry incubations (Bell et al., 2018; Chakraborty et al., 2018).

All diversity analyses were conducted using the phyloseq package (McMurdie and Holmes, 2013) within the R software environment version 3.4.2 (R Development Core Team., 2010). Community similarities were measured using the weighted UniFrac distance matrices (Lozupone and Knight, 2005) and were visualized using the Principal Coordinates biplot using the function “ordinate” from phyloseq.

### Metabolomic analysis

For the analysis of metabolites, a 0.5 ml slurry subsample was centrifuged at 21,000 × g for 10 min at room temperature. The supernatant was diluted 1:1 with pure methanol and subsequently filtered through Teflon syringe filters (0.22 μm pore size) to remove any remaining suspended particles. Metabolites present in the extract were separated with UHPLC using a gradient of 20 mM ammonium formate at pH 3.0 in water (solvent A) and 0.1% formic acid (% v/v) in acetonitrile (solvent B) in conjunction with a Synchronis™ HILIC LC column (100 mm × 2.1 mm × 2.1 μm; Thermo Scientific, CA, USA). High-resolution mass spectra were acquired on a Thermo Scientific Q-Exactive™ HF Hybrid Quadrupole-Orbitrap mass spectrometer coupled to an electrospray ionization source. Data were acquired in negative ion full-scan mode from 50 to 750 *m*/*z* at 240,000 resolution with automatic gain control (AGC) target of 3e6 and a maximum injection time of 200 ms. Untargeted metabolites were analyzed using MAVEN software (Melamud et al., 2010). Metabolites were assigned based on accurate mass and retention times of observed signals relative to standards (where available).

### Enrichment and isolation of sulfate-reducing bacteria (SRB)

Smeerenburgfjorden sediment was used to set up multiple slurries amended with an artificial seawater medium containing sulfate (20 mM). The slurries were supplemented with one of the following substrates: acetate (10 mM), butyrate (5 mM), ethanol (10 mM), formate (10 mM), lactate (10 mM), propionate (10 mM), succinate (10 mM), and H_2_ (with 2 mM acetate). All slurries were incubated at 50°C until cells were observed microscopically, after which aliquots were successively transferred to tubes containing fresh media to dilute away the sediment. Once enrichment cultures were sediment-free, they were used to inoculate 3% agar shake tubes (Widdel and Bak, 1992). Colonies from the agar shakes were picked and further grown in artificial seawater media supplemented with the same substrate they were isolated on.

Cultures from each bacterial strain (30 ml) were centrifuged at 21,000 × g for 10 min. Genomic DNA was extracted from the precipitated biomass using the FastDNA Spin Kit (MP Biomedicals, CA, USA), followed by amplification of the 16S rRNA gene using PCR primers 27F and 1492R (O'Sullivan et al., 2015). PCR products were purified (GenElute™ PCR Clean-Up kit, Sigma-Aldrich) and sequenced along both strands on an ABI3130xl sequencer (Applied Biosystems, CA, USA).

### Phylogenetic analyses

Representative ASV sequences were automatically aligned using the web-based SINA aligner (Pruesse et al., 2012) and imported into the ARB-SILVA database SSU Ref NR 132 (Quast et al., 2013) within the ARB software package (Ludwig et al., 2004). A maximum likelihood (PhyML) tree was calculated using 16S rRNA sequences from the four SRB strains isolated in this study, closely related reference bacteria, and environmental clones based on 1,046 alignment positions by using positional variability and termini filters for bacteria. Using the ARB Parsimony tool, the short ASV sequences were added to this tree by applying the 50% sequence conservation filter and positional variability filters covering the length of the representative sequences without changing the overall tree topology.

### ^35^S-sulfate reduction rates

SRRs in the sediment slurries were measured as described previously (Hubert et al., 2010). In brief, duplicate slurry aliquots sub-sampled at each time interval from a non-radioactive experimental slurry bottle were incubated in 15-ml Hungate tubes injected with ~100 kBq ^35^S-labeled carrier-free sulfate tracer and sealed with butyl rubber stoppers. Following 1 to 2 h of incubation at 50°C in parallel with the non-radioactive slurry, sulfate reduction was terminated by injecting zinc acetate (20% w/w) into the Hungate tubes, followed by storage at −20°C. This was performed 18 separate times at regular intervals throughout a 126-h incubation period to reveal sulfate reduction dynamics as a function of incubation time. SRRs in the slurry aliquots were determined using a cold chromium distillation method as described elsewhere (Kallmeyer et al., 2004).

## Results and discussion

### Different metabolic responses in heated marine sediments

**Figure 1:**
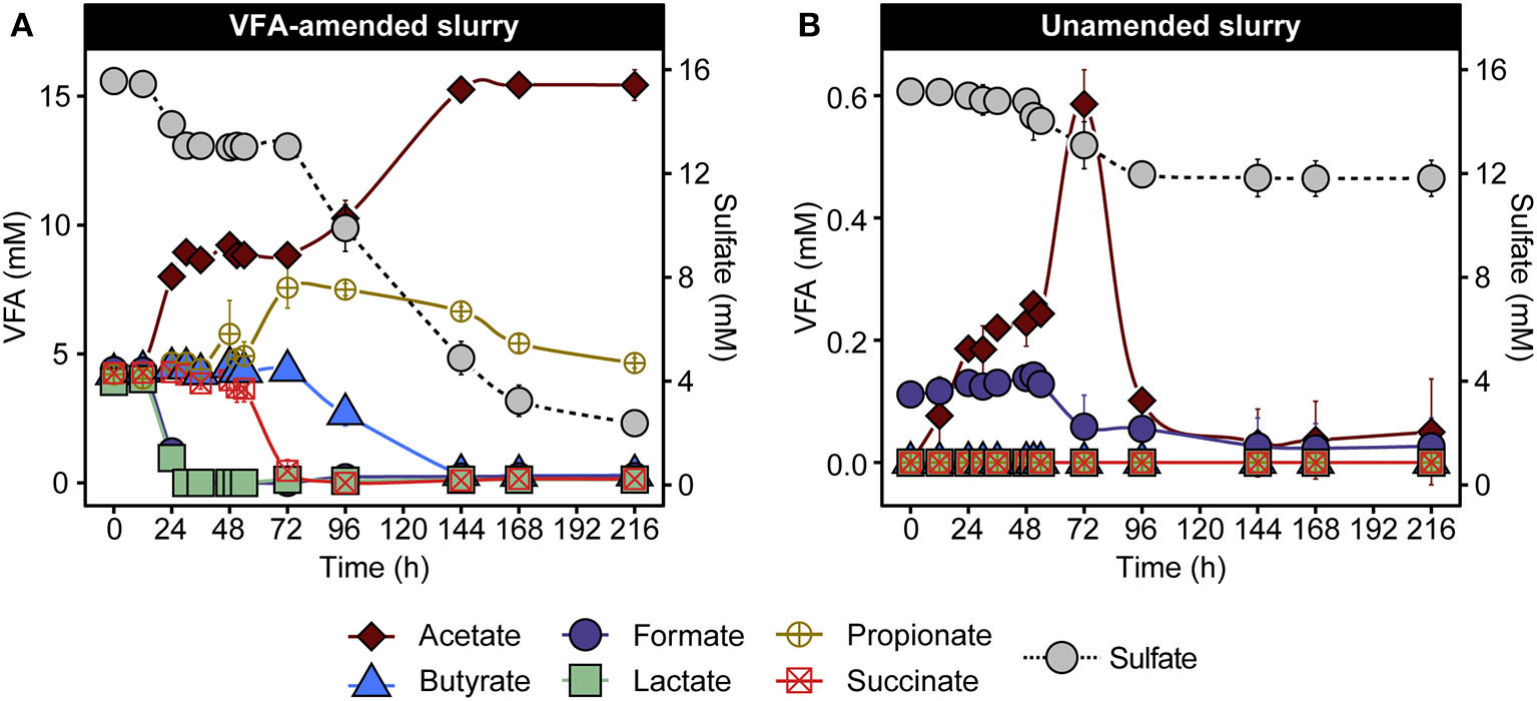
VFA and sulfate concentrations in VFA-amended **(A)** and unamended **(B)** sediment slurry incubations at 50°C. Data are presented as mean ± the standard deviations from three replicate slurries. When not shown, the error bars are smaller than the symbols. Note different scales in the left y-axis in **(A,B)** owing to the different initial concentrations of VFA.

To investigate microbial community response in laboratory incubations, triplicate anoxic slurries consisting of cold Arctic marine sediment supplemented with and without six volatile fatty acids (VFAs) were incubated for 216 h in the dark at 50°C. VFA amendment provides electron donors for spore-forming SRB and has been used similarly in previous studies (Hubert et al., 2010; de Rezende et al., 2013; Müller et al., 2014; Bell et al., 2018; Chakraborty et al., 2018; Cramm et al., 2019; Hanson et al., 2019; Gittins et al., In Press). Changes in VFA and sulfate concentrations were observed in both VFA-amended and unamended sediment slurries (Figure 1). In the VFA-amended slurries, lactate and formate were depleted first (between 0 and 30 h), followed by depletion of succinate and simultaneous accumulation of propionate between 48 and 72 h, followed by consumption of propionate and butyrate between 72 and 216 h (Figure 1A). A cumulative increase in acetate to 11.2 ± 0.6 mM occurred in two distinct phases between 12 and 30 h and 72 and 144 h in the VFA-amended slurries. Acetate accumulation was also observed in the unamended slurries, but to a lesser extent, increasing from background levels up to 0.6 mM (Figure 1B). Sulfate depletion was more extensive and pronounced in VFA-amended slurries, decreasing from 15.6 ± 0.3 mM to 2.5 ± 0.1 mM during the incubation period (Figure 1A), occurring in two phases, similar to the acetate accumulation. By comparison, sulfate concentrations in the unamended slurries dropped from 15.16 ± 0.2 mM to a final concentration of 12.0 ± 0.7 mM over the same 216 h incubation period (Figure 1B).

These patterns of VFA and sulfate concentrations are consistent with previous demonstrations of metabolic activities catalyzed by thermophilic endospore communities in heated sediments from various fjords within the Svalbard archipelago (Hubert et al., 2009; Hanson et al., 2019), suggesting the activation of anaerobic thermophiles including fermentative and respiratory (i.e., sulfate-reducing) populations. In the VFA-amended slurries, acetate accumulation was likely driven by the incomplete oxidation of VFAs coupled with sulfate reduction (Muyzer and Stams, 2008), based on the biphasic drop in sulfate and corresponding increases in acetate (Figure 1A). Consumption of sediment organic matter likely also contributed toward net acetate accumulation, as observed in slurries not amended with VFAs (Figure 1B), where acetate accumulation was observed as early as 12 h and preceded sulfate depletion. Against the large background of VFAs in the amended incubation, fermentation of sediment organics into smaller by-products in the initial hours of incubation is harder to discern. Depletion of the amended succinate coupled with a stoichiometric increase in propionate between 54 and 72 h did not coincide with either of the two sulfate reduction phases described above (Figure 1A), which is suggestive of a fermentative decarboxylation reaction (Janssen et al., 1996). Overall, VFA and sulfate measurements point toward the heat-induced proliferation of fermentative and sulfate-reducing thermophiles present as dormant spores in permanently cold Arctic marine sediments.

### Succession of thermophilic endospore communities in slurry incubations

**Figure 2:**
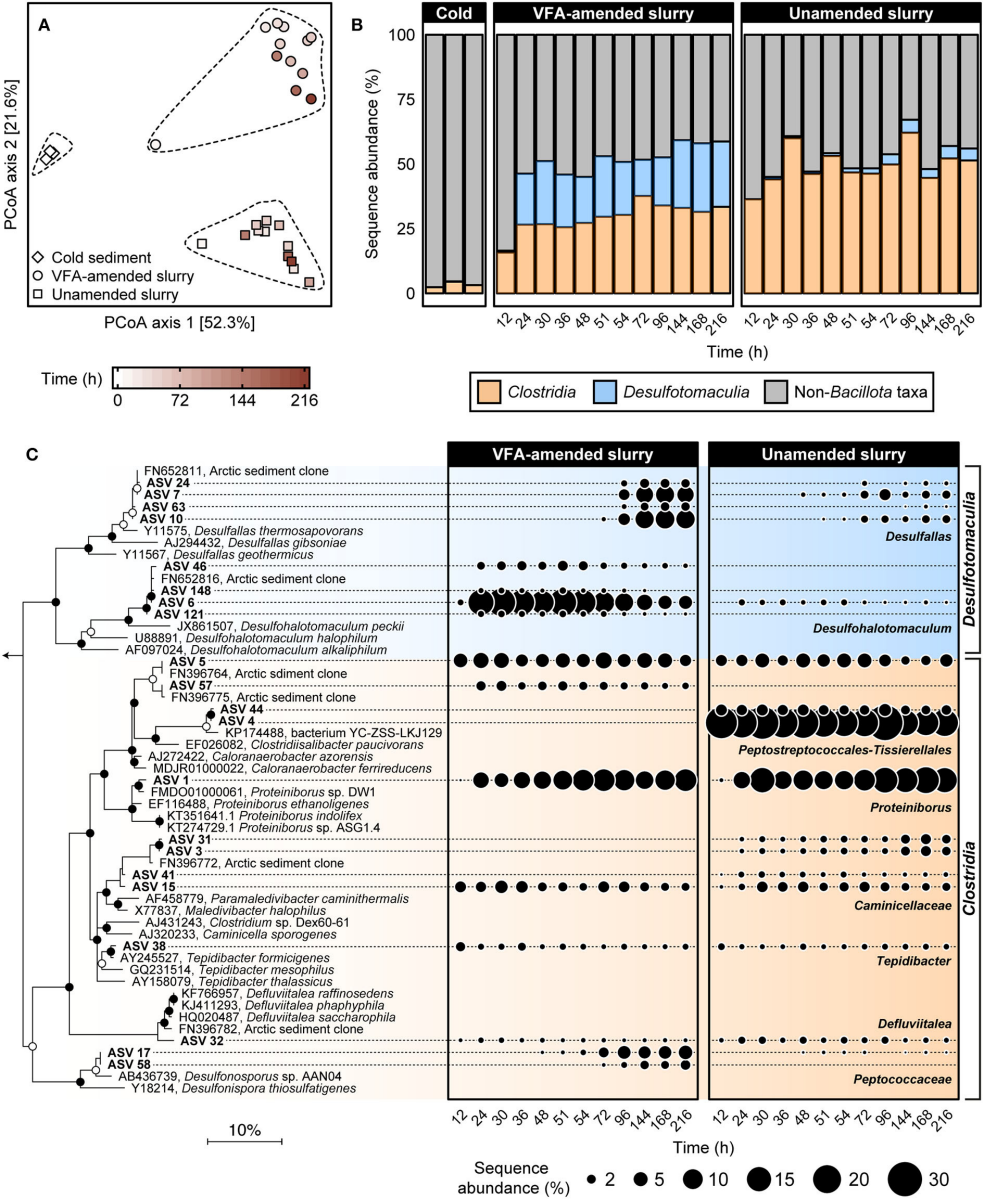
Comparison of bacterial community similarity in cold sediments before and after heating with or without VFA amendment based on weighted UniFrac distances **(A)**. The 27 different 16S rRNA gene amplicon libraries were randomly subsampled to 16,667 reads to account for unequal sequencing depth across libraries and to ensure comparability of sample diversity. Heating to 50°C led to an enrichment in relative sequence abundance of bacterial classes *Clostridia* and *Desulfotomaculia* within the phylum *Bacillota* over a 12–216 h time period **(B)**. Relationships among 21 spore-forming bacterial ASVs enriched in slurry incubations together with close relatives (>96% sequence identity) are shown in the maximum likelihood phylogenetic tree **(C)**. Filled and open circles at branch nodes indicate lineages with >80% and 50–80% bootstrap support, respectively, based on 1,000 re-samplings. The scale bar represents 10% estimated sequence divergence as inferred from maximum likelihood analysis. *Geobacter metallireducens* (NCBI accession L07834; not shown) were used as an outgroup. Bubble plots show percent relative sequence abundance and succession of 21 *Bacillota* ASVs enriched in the heated slurry incubations. Sequence abundances of Non-*Bacillota* taxa are shown in Supplementary Figure 1.

To investigate the succession of thermophilic bacteria in high-temperature laboratory incubations, 16S rRNA gene amplicon libraries were established before and throughout both incubation experiments. Principal coordinate analysis of weighted UniFrac distances comparing bacterial community similarities revealed a pronounced community shift after pasteurization and over time at 50°C (Figure 2A). This corresponds to a clear increase in the sequence abundance of the phylum *Bacillota* (formerly known as *Firmicutes*; Oren and Garrity, 2021), to which all known endospore-forming bacteria belong. This was already evident after 12 h of incubation (Figure 2B), and within 24 h, members of the classes *Clostridia* and *Desulfotomaculia* comprised ca. Forty-six percent of sequence reads in both VFA-amended and unamended slurries and remained dominant throughout the 216 h incubations. *Clostridia* represented on average 30.5 ± 3.8% and 50.6 ± 6.1% of the community between 24 and 216 h in the VFA-amended and unamended slurries, respectively. *Desulfotomaculia* is a new class originating from the recent taxonomic reorganization within *Bacillota* (Parks et al., 2018) consisting of spore-forming, sulfate-reducing bacteria (Aüllo et al., 2013). *Desulfotomaculia* were on average 21.5 ± 3.98% in the VFA-amended slurries, compared to only 2.67 ± 1.72% in the unamended slurries (Figure 2B). This is consistent with the extent of sulfate reduction activity being 4-fold greater in the slurries amended with VFA. The high sequence abundance of *Desulfotomaculia* in the VFA-amended slurries likely contributed toward the community dissimilarity between VFA-amended and unamended slurries (Figure 2A). Six other bacterial phyla, namely *Actinobacteriota, Bacteroidota, Campylobacterota, Cyanobacteria, Desulfobacterota*, and Pseudomonadota (formerly *Proteobacteria*) cumulatively represented almost 75% of the bacterial communities in the cold sediments before incubation. The sequence abundances of each of these phyla noticeably decreased in the heated slurries (Supplementary Figure 1), as members of the *Bacillota* became enriched (Figure 2B).

Twenty-one *Bacillota* ASVs were identified as increasing in sequence abundance across all amplicon libraries during incubation at 50°C (Supplementary Table 1). Fifteen out of these 21 ASVs were detected within 24 h of incubation and were present in all subsequent subsampling intervals (Figure 2C). Eleven of these 15 early ASVs represented family-level and genus-level groups within *Clostridia* including known obligately fermentative lineages, for example, *Caloranaerobacter, Caminicella, Clostridiisalibacter, Defluvitalea, Proteiniborus*, and *Tepidibacter*. The other four early ASVs all belong to the newly proposed genus *Desulfohalotomaculum* within the *Desulfotomaculia*. Among these, the sequence abundance of ASV 6 (*Desulfohalotomaculum*) increased from <1 to >15% between 12 and 24 h in the VFA-amended slurries (Figure 2C). The other three *Desulfohalotomaculum* ASVs were detected at much lower sequence abundances and were not detected in the unamended slurry libraries (Figure 2C). Among the six ASVs that were detected in later subsampling intervals, four belonged to the renamed genus *Desulfallas* (ASVs 7, 10, 24, and 63) within the *Desulfomaculia*. Sequence abundances of ASVs 7 and 10 increased noticeably between 72 and 144 h especially in the VFA-amended incubations. Two ASVs belonging to the family *Peptococcaceae* (ASV 17 and 58) increased in the VFA-amended slurries between 48 and 96 h, concurrent with the onset of succinate depletion and propionate accumulation.

ASV 1, belonging to the genus *Proteiniborus*, was detected in both VFA-amended and unamended slurries within 24 h of incubation and represented 6% to 15% of the community between 12 and 216 h (Figure 2C). This genus is frequently detected in anoxic, organic-rich environments such as anaerobic sludge digesters (Maspolim et al., 2015; Dai et al., 2016; Zhang et al., 2016; Wu et al., 2017). Type strains representing two different species within *Proteiniborus* were isolated from a laboratory-scale up-flow anaerobic sludge reactor (Niu et al., 2008) and an industrial-scale biogas fermenter (Hahnke et al., 2018). Fermentative growth by these isolates is strongly stimulated by peptone and a mixture of amino acids as carbon sources, compared to much less stimulation when supplied with simple sugars. Both strains produce acetate along with CO_2_ and H_2_ during fermentative growth on proteins (Hahnke et al., 2018). ASV 4, belonging to the genus *Clostridiisalibacter*, represented 24% starting at 12 h in the unamended slurries but was not detected in the VFA-amended slurries (Figure 2C). The type strain within this genus is capable of fermenting certain amino acids (Liebgott et al., 2008). Early enrichment of *Clostridiisalibacter* was observed in Aarhus Bay marine sediments incubated at high temperatures (Volpi et al., 2017). Two other ASVs observed within the early hours of incubation belonged to *Tepidibacter* and *Defluviitalea*, both known for their ability to ferment carbohydrates (Urios et al., 2004; Jabari et al., 2012; Ma et al., 2017). In particular, ASV 32 is a close relative (>99% sequence identity) to *Defluviitalea phaphyphila*, a thermophile capable of decomposing brown algal biomass and producing acetate and pyruvate (Ji et al., 2015).

Taken together, the emergence of members of the above genera in sequence libraries and accumulation of acetate in the early hours of incubation demonstrated an ecological succession of metabolically distinct thermophilic endospores as a result of heat induction where fermentative populations were established earlier than sulfate-reducing populations. Laboratory-engineered microbial communities have previously been used to study community succession (Callbeck et al., 2011; Cai et al., 2019; Vick et al., 2019) and heat-induced endospore communities, therefore, present a good model system for investigating microbial community dynamics as a result of sudden environmental perturbations.

### Production and consumption of amino acids in early hours of incubation

**Figure 3:**
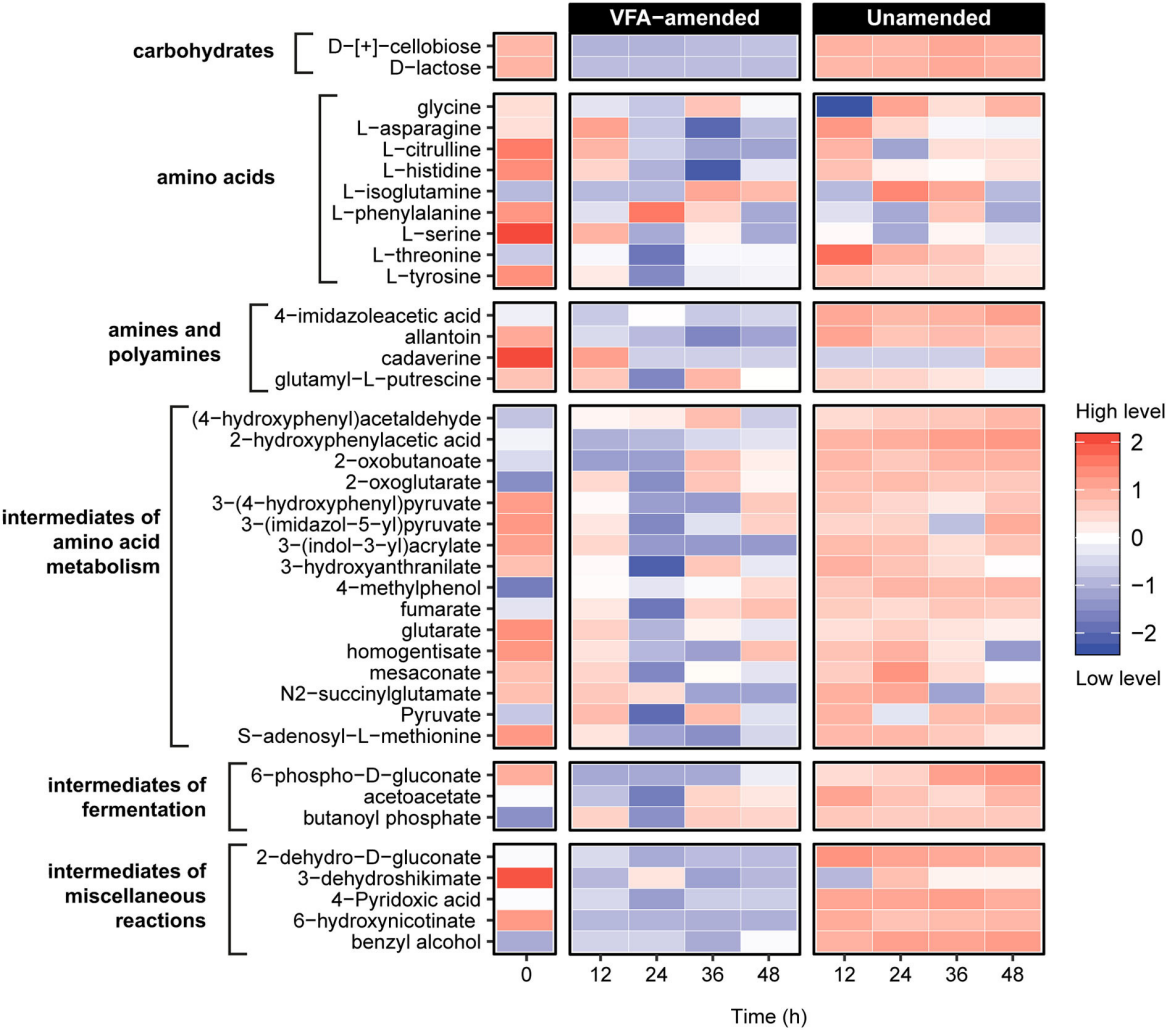
Untargeted metabolite analysis using UHPLC Orbitrap mass spectrometry revealed 39 compounds related to organic matter decomposition in sediment heating incubations. Metabolite levels at hours 12–48 are expressed as the logarithmically normalized mean fractional abundance of technical replicates (*n* = 5). The time zero (after pasteurization) column represents average metabolite levels of VFA-amended (*n* = 3) and unamended (*n* = 3) slurries. A larger heatmap specifically showing amino acids and intermediate compounds of amino acid metabolism reactions are presented in Supplementary Figure 3.

To further investigate the metabolic reactions catalyzed in the early hours of incubation, untargeted metabolomic analyses using liquid chromatography coupled with Orbitrap mass spectrometry were conducted on slurry subsamples representing five intervals within the first 48 h of incubation (Figure 3). Unsupervised principal component analysis of the metabolite profiles constructed based on a set of 79 identified compounds revealed a statistically significant clustering of the samples before (0 h) and after (12–48 h) incubation within VFA-amended and unamended sediment slurries (Supplementary Figure 2; Supplementary Table 2). Distinct metabolite profiles of VFA-amended and unamended slurries indicate that the external addition of VFAs as an instant energy source influences the metabolic processes catalyzed by resuscitated thermophilic endospore populations.

Nine amino acids and numerous compounds assigned as pathway intermediates of amino acid degradation were identified in both VFA-amended and unamended slurries (Figure 3; Supplementary Figure 3). In general, amino acid metabolites were much more abundant in the unamended samples. Levels of L-citrulline, L-histidine, and L-serine decreased consistently within 24 h of incubation in unamended slurries, while in the VFA-amended slurries, levels of all three amino acids first increased until 12 h and then decreased in subsequent sampling points. A similar pattern was observed for L-asparagine and L-threonine in unamended slurries. This could mean that these amino acids were likely produced *via* protein and peptide breakdown followed by subsequent metabolic assimilation. Among potential derivatives of amino acid degradation, levels of fumarate and pyruvate were observed to fluctuate, indicating production as well as consumption at different points during the first 48 h of incubation. Levels of 2-oxoglutarate, an important intermediate for various central metabolic pathways and amino acid fermentation *via* Stickland reactions, increased in all slurries. Overall, these observations demonstrate that the breakdown of proteins, peptides, and amino acids was catalyzed in the early hours of the incubations, consistent with the emergence of putative protein-fermenting thermophiles such as *Proteiniborus* and *Clostridiisalibacter* identified in the 16S rRNA gene libraries.

### Distinct sulfate reduction responses from early and late germinating SRB populations

Sulfate and VFA depletion patterns observed in the heated sediment slurries (Figure 1) and the appearance of two SRB genera in the 16S rRNA gene libraries in the early and late hours of the VFA-amended incubation (Figure 2) suggested that sulfate reduction was catalyzed by metabolically distinct sulfate-reducing populations during two distinct phases. To further examine the metabolic diversity of SRB in Smeerenburgfjorden sediments, four pure cultures (strains Eth-2, For-1, Lac-2, and Hyd-1) were isolated using a serial dilution of sediment slurries followed by colony picking from agar shake tubes. Three out of these four strains (strain Hyd-1 was isolated on H_2_) demonstrated ability for organotrophic sulfate reduction as confirmed by monitoring sulfate depletion in sediment-free liquid cultures as well as by measuring SRR with ^35^S radiotracer. Phylogenetic analysis using near-full-length 16S rRNA gene sequences revealed that strains Lac-2 and Hyd-1 belonged to the genus *Desulfohalotomaculum* while strains Eth-2 and For-1 are members of the genus *Desulfallas* (Supplementary Figure 4). Furthermore, *Desulfotohalotomaculum* strain Lac-2 and ASV 6 from the incubation experiment share >99% 16S rRNA gene sequence identity. In the *Desulfallas* group, ASV 7, ASV 10, strain For-1, and strain Eth-2 share >99% sequence identity with each other.

Physiological characterization of the SRB isolates corroborates the biphasic sulfate and VFA dynamics observed in slurry incubations and the enrichment of *Desulfohalotomaculum* and *Desulfallas* populations at different intervals in VFA-amended incubations. *Desulfohalotomaculum* Strain Lac-2 couples sulfate reduction to the oxidation of lactate and formate whereas *Desulfallas* strain For-1 oxidizes butyrate and propionate (Supplementary Table 3). These isolate phenotypes suggest that distinct phases of sulfate reduction between 12 and 30 h and 72 and 168 h (Figure 1A) in the slurry incubations were catalyzed by *Desulfohalotomaculum* oxidizing lactate and formate, and by *Desulfallas* oxidizing butyrate and propionate, respectively. This biphasic nature of these responses suggests that *in situ* abundance and/or germination timing of these two populations of spore-forming SRB may be different.

**Figure 4:**
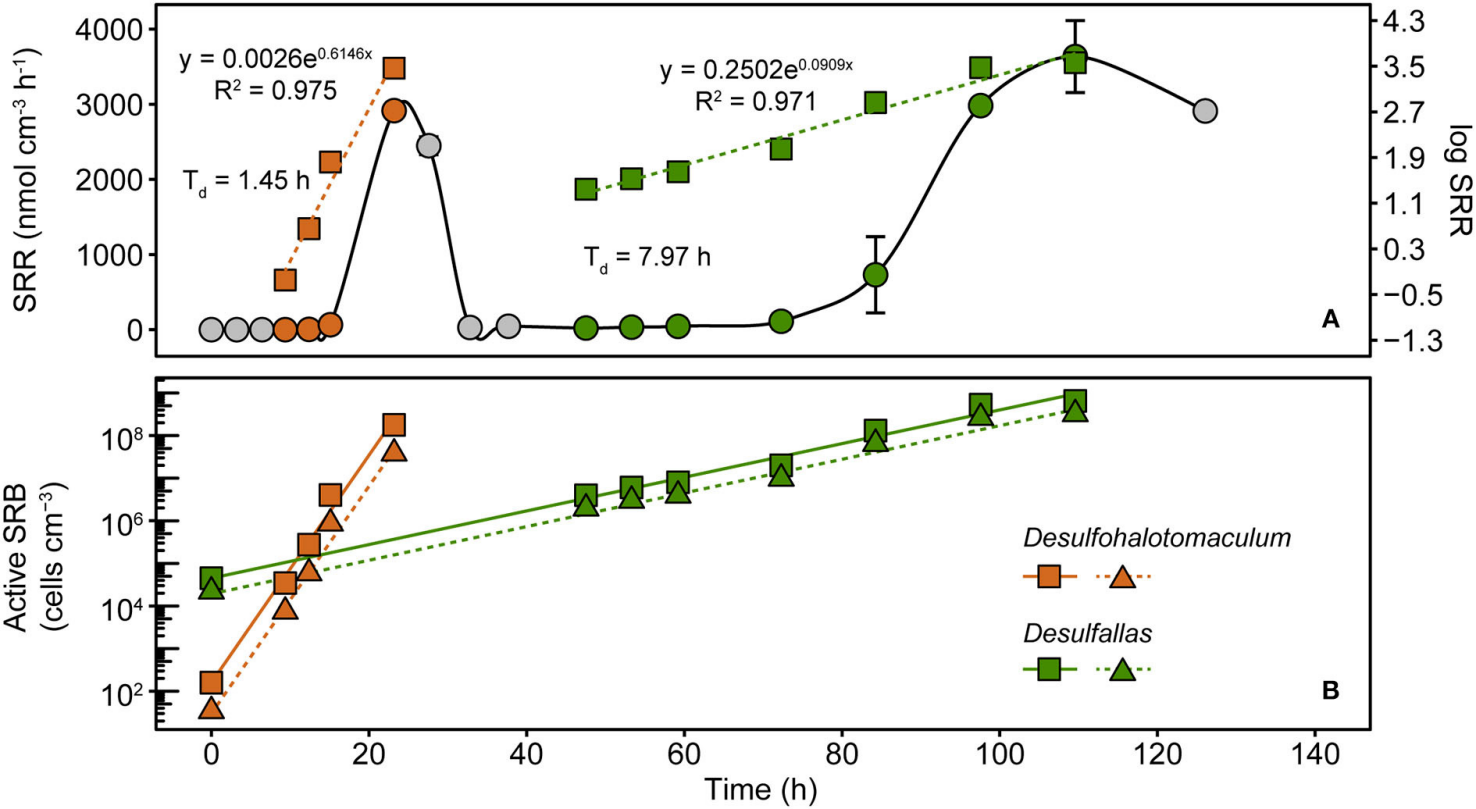
Sulfate reduction rates (SRRs) catalyzed by populations of sulfate-reducing thermophiles enriched in sediments heated to 50°C. SRRs are plotted as an average of duplicate measurements from discreet subsampling intervals **(A)**. Exponential increases in SRR were observed in two distinct time intervals, between 9 and 23 h and 47 and 110 h. These two phases are evident when plotting the same data on linear (circles; left y-axis) and logarithmic (squares, right y-axis) scales. SRR enabled cell abundances to be estimated for two thermophilic, spore-forming SRB populations belonging to *Desulfohalotomaculum* and *Desulfallas* **(B)**. For both populations, two different assumptions for the biovolume of a single SRB cell were used to estimate abundance. Squares denote SRB estimates based on cellular biovolume of 1.0 pg μm^−3^, the median determined by comparing 68 different SRB strains (de Rezende et al., 2017). Triangles denote SRB estimates that assume cellular biovolumes for the corresponding populations determined from cellular morphologies of two SRB strains isolated in this study. Solid and dashed lines represent linear smoothers fitted onto the symbols for each set of estimated cell numbers. Doubling times shown next to the estimated abundances were calculated from the exponential equations for increasing SRR.

To assess these dynamics in greater detail, marine sediment from Smeerenburgfjorden was incubated at 50°C with ^35^S radiotracer incorporation. SRRs were measured during the first 126 h to capture the two sulfate reduction responses shown in Figure 1. Distinct exponential increases in sulfate reduction were observed during early (9–23 h) and late (47–110 h) intervals (Figure 4A), consistent with biphasic sulfate decrease in Figure 1A. SRRs were subsequently used to estimate doubling times and *in situ* (i.e., time-zero) cell densities of the two SRB populations (Hubert et al., 2009; de Rezende et al., 2017). Exponential functions derived from increases in SRR revealed that the first phase of sulfate reduction was catalyzed by a rapidly growing SRB population (presumably *Desulfohalotomaculum*; Figure 3C) with a doubling time of 1.5 h, whereas the second phase was catalyzed by a slower 8.0 h doubling time catalyzed by a second SRB population (presumably *Desulfallas*; Figure 3C). Further extrapolating these results and assuming the onset of sulfate reduction corresponds to the germination of *Desulfohalotomaculum* and *Desulfallas* at the beginning of the incubation (0 h), the early and the late responses correspond to stimulation of *in situ* populations that are 1.6 × 10^2^ and 4.5 × 10^4^ endospores cm^−3^, respectively. These estimations assume constant biomass for a single cell biovolume (1.0 pg μm^−3^) as described previously (de Rezende et al., 2017). Alternatively, when biomass per cell was estimated based on cellular morphologies of the *Desulfohalotomaculum* and *Desulfallas* strains isolated here (Supplementary Figure 4; Supplementary Table 3), the estimated *in situ* abundances were slightly lower (Figure 4B). Despite an *in situ* abundance of spores that is estimated to be nearly two orders of magnitude lower, the fast-growing *Desulfohalotomaculum* lineage responds quickly to the increase in temperature and the presence of formate and lactate, with the slower-growing *Desulfallas* cells oxidizing propionate and butyrate later in the incubations.

## Conclusion

Heating Arctic marine sediment samples demonstrate microbial community re-organization that occurs during dramatic environmental upheaval events. Incubating sediments at a temperature much warmer than ambient conditions, with and without labile substrates, triggered a cascade of changes in the community composition of germinated spore-forming thermophiles. Differences in diversity and metabolism were influenced by the VFA amendment. Earlier and later responses by different community members revealed by 16S rRNA gene sequencing were accompanied by metabolic activities that included early fermentation of amino acids and other sediment organics, and distinct SRB populations using different fermentation products as electron donors. Previous research has shown that many of these misplaced thermophile populations have likely dispersed from warm habitats such as deep petroleum reservoirs or crustal fluids discharging from mid-ocean ridges. The ability to retain viability through dormancy under cold marine conditions coupled with a physiological capability to grow rapidly at high temperatures in a concerted way likely equips these bacterial populations to germinate and establish as important community constituents in the event of dramatic perturbations. The results suggest *in situ* abundance and nutrient availability including *via* metabolic cross-feeding contribute toward the ecological dynamics between newly activated microbial populations in these situations.

## Data availability statement

The datasets presented in this study can be found in online repositories. The names of the repository/repositories and accession number(s) can be found below: https://www.ncbi.nlm.nih.gov/, BioProject PRJNA843862; https://www.ncbi.nlm.nih.gov/, JQ304694-JQ304697.

## Author contributions

AC, JR, BJ, and CH contributed to the conceptualization of the study, funding acquisition, experimental design, conducting experiments, data analyses, writing, and editing the manuscript. SD, SM, and CL helped conduct experiments and analyze data. All authors contributed to manuscript revision and approved the submitted version.

## Funding

Metabolomics data were acquired at the Calgary Metabolomics Research Facility (CMRF), which was supported by the International Microbiome Centre and the Canada Foundation for Innovation (CFI-JELF 34986). We acknowledge additional funding and support from Mitacs, NSERC, Genome Canada, the Government of Alberta, the Max Planck Society, and the Danish Research Foundation.

## Conflict of interest

The authors declare that the research was conducted in the absence of any commercial or financial relationships that could be construed as a potential conflict of interest.

## Publisher's note

All claims expressed in this article are solely those of the authors and do not necessarily represent those of their affiliated organizations, or those of the publisher, the editors and the reviewers. Any product that may be evaluated in this article, or claim that may be made by its manufacturer, is not guaranteed or endorsed by the publisher.
