# Supplementary material for: Metabolic responses of thermophilic endospores to sudden heat-induced perturbation in marine sediment samples
Source: Front Microbiol. 2022 Aug 12;13:958417. doi: 10.3389/fmicb.2022.958417 (PMC9411986; doi:10.3389/fmicb.2022.958417)
Supplement: Supplementary file 1 [file Data_Sheet_1.DOCX]

Supplementary Figure 1. Sequence abundance of non-*Bacillota* taxa in 16S rRNA gene amplicon libraries representing cold Arctic sediments and heated sediment slurries. Fourteen bacterial phyla are shown which were detected at >1 % sequence abundance in at least one library. The Others category cumulatively represents taxa that were always detected at < 1% sequence abundance.


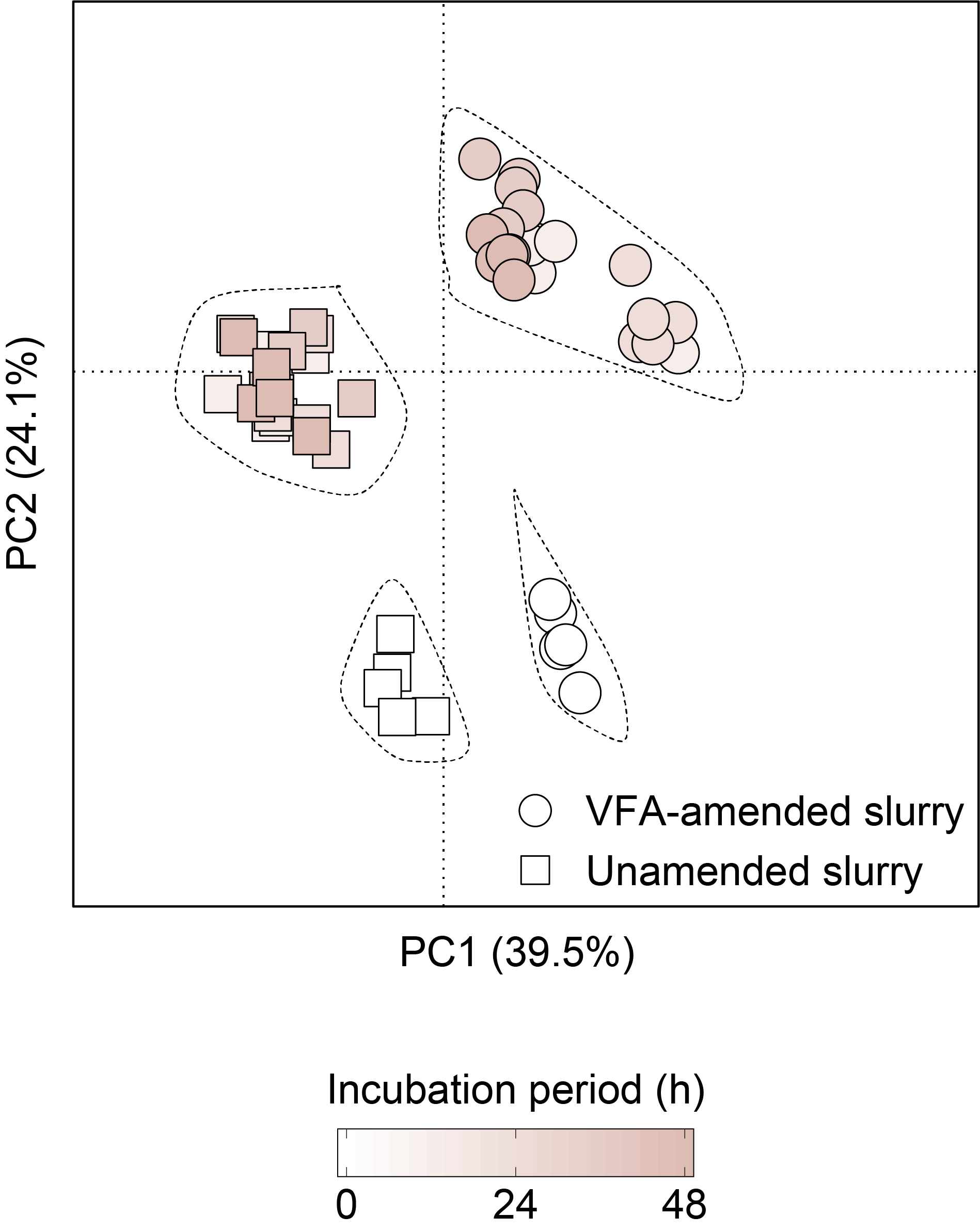


Supplementary Figure 2. Principal component analysis of untargeted metabolite profiles obtained from five subsampling time points (0-48 h; technical replicates / time point = 5) of VFA-amended and unamended sediment slurries. Each sample is represented by 79 identified metabolites.


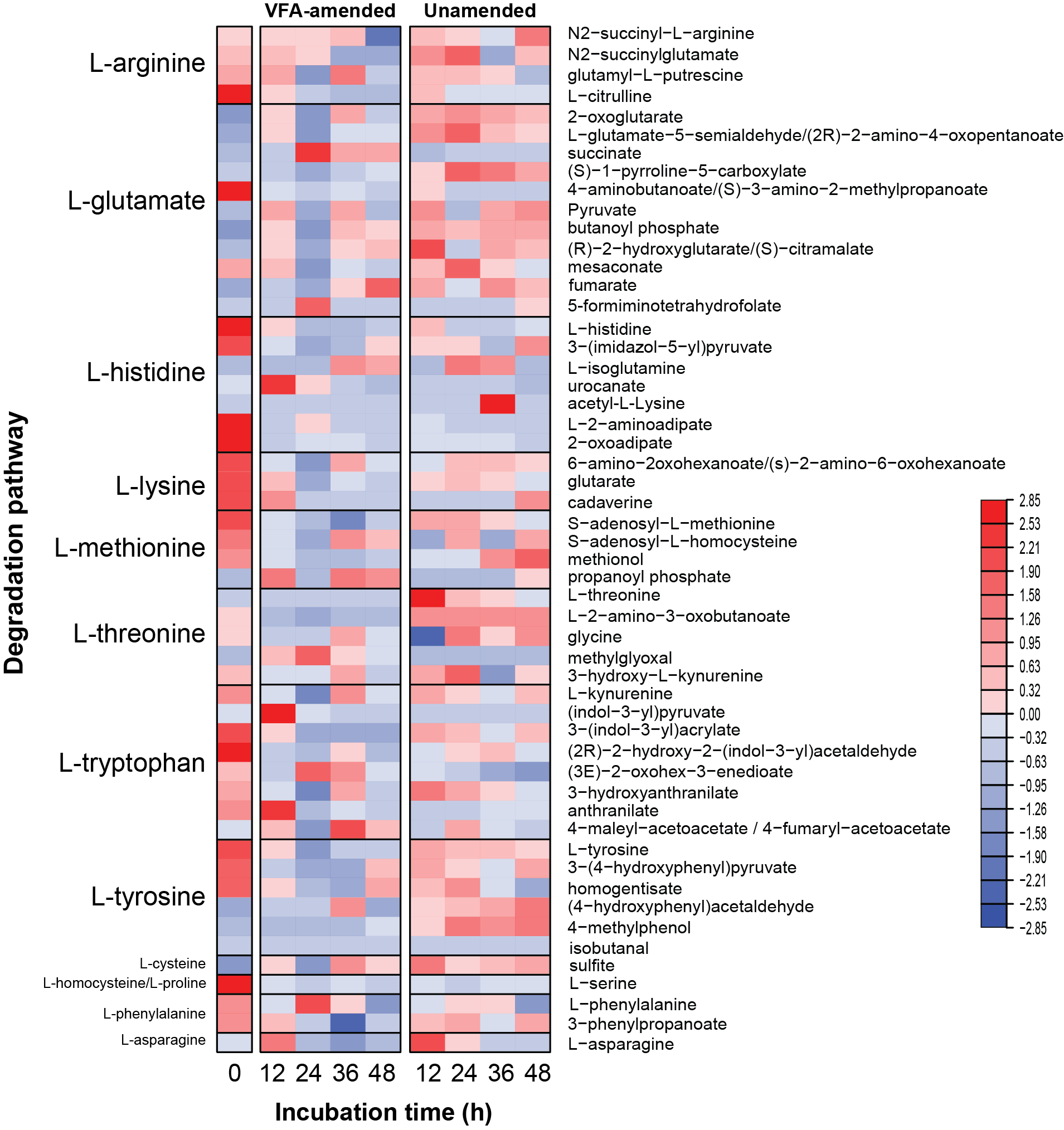


Supplementary Figure 3. Untargeted metabolite analysis using UHPLC Orbitrap mass spectrometry showing metabolites related to amino acid metabolism identified in sediment slurries. Metabolite levels at hours 12-48 are expressed as logarithmically normalized mean fractional abundance of technical sample replicates (n = 5). The time zero (after pasteurization) column represents average metabolite levels of three VFA-amended and three unamended slurries. Metabolic pathway assignments are indicated on the left of the heat map and corresponding compound names are listed on the right.


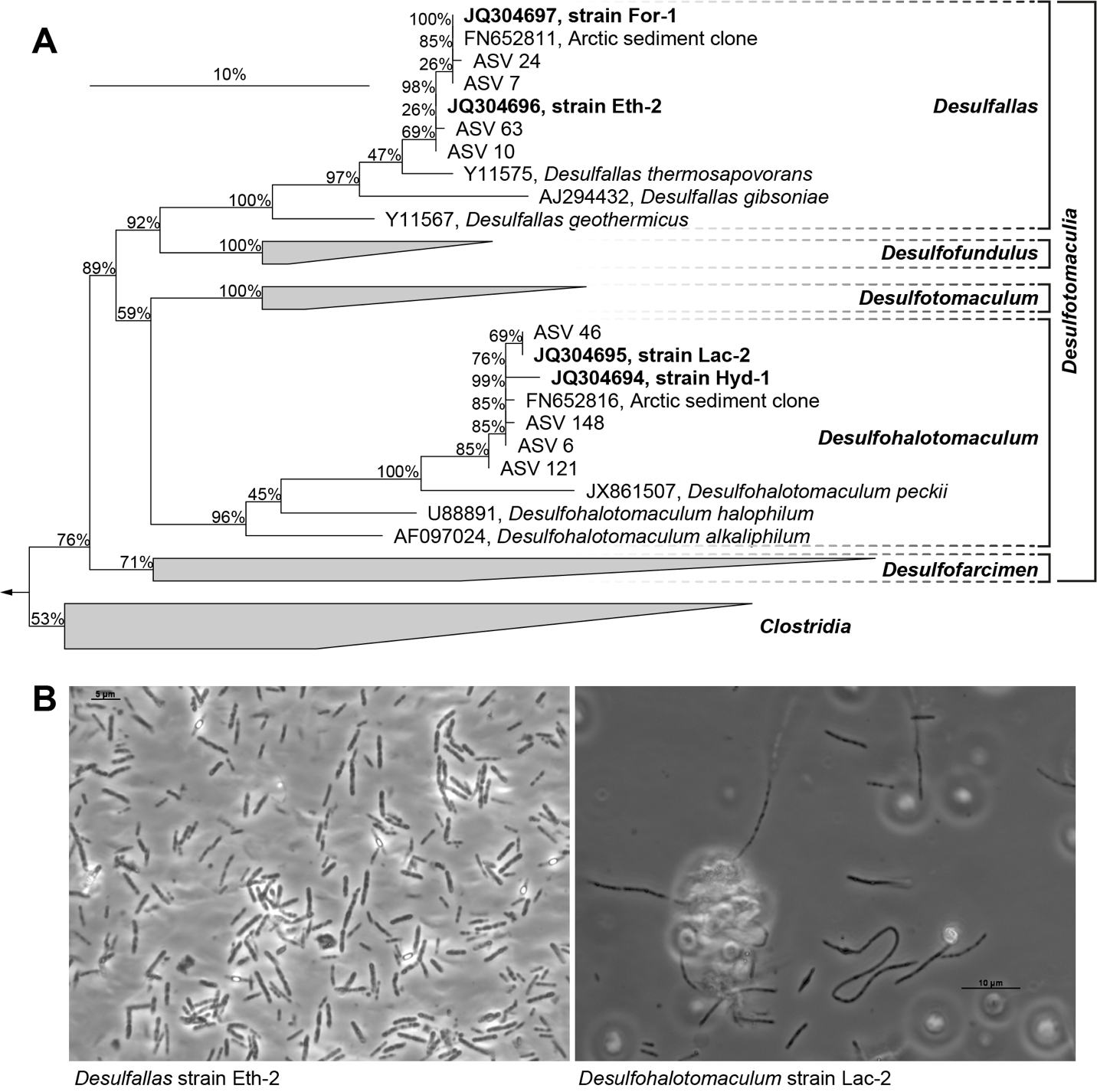


Supplementary Figure 4: 16S rRNA-based phylogenetic tree (panel A) showing four sulfate-reducing bacterial strains (in bold) isolated in this study along with their phylogenetic relatives (>96% sequence identity). Closely related sequences include eight *Desulfotomaculia* ASVs identified in the slurry incubations. Percent values at the node of the branches indicate bootstrap support based on 1000 re-samplings. Boxes show different genus-level clades. The scale bar represents 10% estimated sequence divergence as inferred from maximum likelihood analysis. *Geobacter metallireducens* (NCBI accession - L07834; not shown) was used as an outgroup to root the tree. Photomicrographs of two isolated SRB strains (Panel B) show the difference in average cell size between *Desulfallas* strain Eth-2 and *Desulfohalotomaculum* strain Lac-2. The oval translucent structures are the endospores.

| Thermospore ASV | Taxonomy assigned by SILVA reference database (version 138) | Next cultured relative | Accession number | Sequence identity |
| --- | --- | --- | --- | --- |
| ASV1 | Bacteria;Firmicutes;Clostridia;Peptostreptococcales-Tissierellales;Proteiniboraceae;Proteiniborus | Proteiniborus indolifex strain BA2-13 | NR_159905.1 | 98.50% |
| ASV3 | Bacteria;Firmicutes;Clostridia;Peptostreptococcales-Tissierellales;Caminicellaceae;Caminicella | *Maledivibacter halophilus* strain DSM 5387 | NR_125713.1 | 96.29% |
| ASV4 | Bacteria;Firmicutes;Clostridia;Peptostreptococcales-Tissierellales;Peptostreptococcales-Tissierellales_fa;Clostridiisalibacter | Bacterium YC-ZSS-LKJ129 | KP174488.1 | 99.25% |
| ASV5 | Bacteria;Firmicutes;Clostridia;Peptostreptococcales-Tissierellales;Peptostreptococcales-Tissierellales_fa;Caloranaerobacter | *Caloranaerobacter* sp. TR13 | KC533829.1 | 97.26% |
| ASV6 | Bacteria;Firmicutes;Desulfotomaculia;Desulfotomaculales;Desulfotomaculales_fa;Desulfohalotomaculum | *Desulfotomaculum* sp. strain Lac-2 | JQ304695.1 | 99.75% |
| ASV7 | Bacteria;Firmicutes;Desulfotomaculia;Desulfotomaculales;Desulfallas-Sporotomaculum;Desulfallas | *Desulfotomaculum* sp. strain For-1 | JQ304695.1 | 100.00% |
| ASV10 | Bacteria;Firmicutes;Desulfotomaculia;Desulfotomaculales;Desulfallas-Sporotomaculum;Desulfallas | *Desulfotomaculum* sp. strain For-1 | JQ304695.1 | 99.75% |
| ASV15 | Bacteria;Firmicutes;Clostridia;Peptostreptococcales-Tissierellales;Caminicellaceae;Caminicella | *Clostridium* sp. DY192 | HQ696463.1 | 97.76% |
| ASV17 | Bacteria;Firmicutes;Clostridia;Peptococcales;Peptococcaceae;Unclassified | *Desulfonosporus* sp. AAN04 | AB436739.1 | 97.66% |
| ASV24 | Bacteria;Firmicutes;Desulfotomaculia;Desulfotomaculales;Desulfallas-Sporotomaculum;Desulfallas | *Desulfotomaculum* sp. strain For-1 | JQ304695.1 | 99.51% |
| ASV31 | Bacteria;Firmicutes;Clostridia;Peptostreptococcales-Tissierellales;Caminicellaceae;Caminicella | *Maledivibacter halophilus* strain DSM 5387 | NR_125713.1 | 96.02% |
| ASV32 | Bacteria;Firmicutes;Clostridia;Lachnospirales;Defluviitaleaceae;Defluviitalea | *Defluviitalea phaphyphila* sp. strain Alg1 | KJ411293.1 | 99.26% |
| ASV38 | Bacteria;Firmicutes;Clostridia;Peptostreptococcales-Tissierellales;Peptostreptococcaceae;Tepidibacter | *Tepidibacter formicigenes* strain L2S1057-4 | JQ670743.1 | 100.00% |
| ASV41 | Bacteria;Firmicutes;Clostridia;Peptostreptococcales-Tissierellales;Caminicellaceae;Caminicella | *Caldanaerocella colombiensis* strain P4.4 | AY464940.1 | 97.77% |
| ASV44 | Bacteria;Firmicutes;Clostridia;Peptostreptococcales-Tissierellales;Peptostreptococcales-Tissierellales_fa;Clostridiisalibacter | Bacterium YC-ZSS-LKJ129 | KP174488.1 | 99.00% |
| ASV46 | Bacteria;Firmicutes;Desulfotomaculia;Desulfotomaculales;Desulfotomaculales_fa;Desulfohalotomaculum | *Desulfotomaculum* sp. strain Lac-2 | JQ304695.1 | 100.00% |
| ASV57 | Bacteria;Firmicutes;Clostridia;Peptostreptococcales-Tissierellales;Peptostreptococcales-Tissierellales_fa;Caloranaerobacter | *Caloranaerobacter* sp. TR13 | KC533829.1 | 97.26% |
| ASV58 | Bacteria;Firmicutes;Clostridia;Peptococcales;Peptococcaceae;Unclassified | *Desulfonosporus* sp. AAN04 | AB436739.1 | 97.43% |
| ASV63 | Bacteria;Firmicutes;Desulfotomaculia;Desulfotomaculales;Desulfallas-Sporotomaculum;Desulfallas | *Desulfotomaculum* sp. strain For-1 | JQ304695.1 | 99.51% |
| ASV121 | Bacteria;Firmicutes;Desulfotomaculia;Desulfotomaculales;Desulfotomaculales_fa;Desulfohalotomaculum | *Desulfotomaculum* sp. strain Lac-2 | JQ304695.1 | 99.50% |
| ASV148 | Bacteria;Firmicutes;Desulfotomaculia;Desulfotomaculales;Desulfotomaculales_fa;Desulfohalotomaculum | *Desulfotomaculum* sp. strain Lac-2 | JQ304695.1 | 99.50% |

Supplementary Table 1. Taxonomy and next cultured relatives of the twenty-one thermospore ASVs that were enriched in slurry incubations at 50ºC

| Broad groups | Comparison groups | F.Model | R^2^ | p.value |
| --- | --- | --- | --- | --- |
| VFA-amended slurry | Unheated (0 h) v Heated (12-48 h) | 17.375 | 0.430 | 0.001 |
| Unamended slurry | Unheated (0 h) v Heated (12-48 h) | 28.494 | 0.553 | 0.001 |
| Unheated sediment | VFA-amended v Unamended | 56.086 | 0.875 | 0.010 |
| Heated sediment | VFA-amended v Unamended | 43.903 | 0.536 | 0.001 |

Supplementary Table 2. Results of Permutational MANOVA

|  |  | SRB strains | | | |
| --- | --- | --- | --- | --- | --- |
|  |  | Lac-2 | Hyd-1 | Eth-2 | For-1 |
| Metabolism (electron donors) | Ethanol | - | - | + | + |
|  | Acetate | - | - | - | - |
|  | Butyrate | - | - | + | + |
|  | Formate | + | - | - | + |
|  | Lactate | + | - | + | - |
|  | Propionate | - | - | + | + |
|  | Succinate | - | - | - | + |
|  | Hydrogen + acetate | + | + | + | + |
| Metabolism (electron acceptors) | No Acceptor | NA | NA | - | + |
|  | Sulfate | + | + | + | + |
|  | Sulfite | NA | NA | + | + |
|  | Thiosulfate | NA | NA | ? | + |
|  | Fumaric acid | NA | NA | - | + |
|  | Elemental sulfur | NA | NA | - | - |
|  | Nitrate | NA | NA | - | + |
|  | Nitrite | NA | NA | - | - |
| Metabolism (disproportionation) | Sulfite | - | NA | + | + |
|  | Thiosulfate | - | NA | - | + |
|  | Fumaric acid | - | NA | - | - |
| Cellular physiology | Cell size (length x width; µm) | 14 x 0.7 | NA | 3.8 x 0.8 | 3.9 x 0.9 |
|  | Morphology | Rod | Rod | Rod | Rod |
|  | Motility | No | No | No | No |
|  | pH range | NA | NA | NA | NA |
|  | Salinity (g/L NaCl) | NA | NA | NA | NA |
|  | Temperature range (°C) | NA | NA | 25-60 | NA |

Supplementary Table 3. Physiological characteristics of the sulfate-reducing bacterial strains
